# Exploring the Dynamics of Virulent and Avirulent Aphids: A Case for a ‘Within Plant’ Refuge

**DOI:** 10.1093/jee/toab218

**Published:** 2021-11-19

**Authors:** Aniket Banerjee, Ivair Valmorbida, Matthew E O’Neal, Rana Parshad

**Affiliations:** 1 Department of Mathematics, Iowa State University, Ames, IA, USA; 2 Department of Entomology, Iowa State University, Ames, IA, USA

**Keywords:** soybean, biotype, insecticide-resistant management, nonlocal ODE model

## Abstract

The soybean aphid, *Aphis glycines* (Hemiptera: Aphididae), is an invasive pest that can cause severe yield loss to soybeans in the North Central United States. A tactic to counter this pest is the use of aphid-resistant soybean varieties. However, the frequency of virulent biotypes that can survive on resistant varieties is expected to increase as more farmers use these varieties. Soybean aphids can alter soybean physiology primarily by two mechanisms, feeding facilitation, and the obviation of resistance, favoring subsequent colonization by additional conspecifics. We developed a nonlocal, differential equation population model to explore the dynamics of these biological mechanisms on soybean plants coinfested with virulent and avirulent aphids. We then use demographic parameters from laboratory experiments to perform numerical simulations via the model. We used this model to determine that initial conditions are an important factor in the season-long cooccurrence of both biotypes. The initial population of both biotypes above the resistance threshold or avirulent aphid close to resistance threshold and high virulent aphid population results in coexistence of the aphids throughout the season. These simulations successfully mimicked aphid dynamics observed in the field- and laboratory-based microcosms. The model showed an increase in colonization of virulent aphids increases the likelihood that aphid resistance is suppressed, subsequently increasing the survival of avirulent aphids. This interaction produced an indirect, positive interaction between the biotypes. These results suggest the potential for a ‘within plant’ refuge that could contribute to the sustainable use of aphid-resistant soybeans.

A challenge for using insect-resistant plants is the evolution of subpopulations of the target pest that survive on these plants. Efforts to limit their frequency can be achieved through the use of insecticide resistance management (IRM) plans (Tabashnik et al. 2013). One subset of pests that are particularly challenging to manage with resistant plants are aphids (Hemiptera: Aphididae). This is due, in part, to their capacity to evolve virulent biotypes that can survive on resistant plants and their remarkable capacity to reproduce asexually across multiple generations (Crowder and Carriere 2009). The ability for some aphids to manipulate their host plant so that subpopulations survive (Elzinga and Jander 2013, Varenhorst et al. 2015a), leaves open the possibility that current models for explaining aphid-plant interactions may not accurately describe unique features of some systems, like that of the soybean aphid, *Aphis glycines* (Hemiptera: Aphididae) on soybeans, *Glycine max* L.

The soybean aphid was first detected in 2000 and has become one of the most important insect pests of soybean in the major production areas of the Midwest United States (Tilmon et al. 2011). It has a heteroecious holocyclic life cycle that utilizes a primary host plant for overwintering and a secondary host plant during the summer. In the spring, aphids emerge and produce three asexual generations on common buckthorn, *Rhamnus cathartica* L., then migrate to soybeans. Aphids continue to reproduce asexually on soybeans, producing as many as 15 generations during the summer (Ragsdale et al. 2011). In North America, aphids arrive on soybean fields beginning in June, where populations increase by four orders of magnitude. At the end of the growing season (September), aphids begin the migration back to their overwintering host, reproduce sexually, and overwinter in the egg stage (Ragsdale et al. 2004). Within Iowa, populations of aphids large enough to reduce soybean yield occurred in 40% of growing seasons from 2004 to 2019, with populations peaking in the middle to the end of August (Dean et al. 2020). Aphid outbreaks can be prevented with insecticides (Ragsdale et al. 2004), however, aphid-resistant varieties of soybean are available and can prevent yield loss without insecticide (Tilmon et al. 2021) but virulent biotypes that can survive on these varieties exist in North America (Hill et al. 2010). The sustainable use of aphid-resistant varieties is not possible without an effort to limit the anticipated increase in virulent biotypes.

Colonization and feeding by an insect herbivore can alter the plant’s physiology, favoring the subsequent colonization of additional conspecifics (Price et al. 2011). There are two mechanisms by which this susceptibility can be induced, feeding facilitation, and the obviation of resistance. Feeding facilitation is a more general mechanism by which the general physiology of the host plant is altered by the herbivore, often in a density-dependent manner. A more specific mechanism that induces susceptibility is the obviation of traits that confer resistance to the herbivore (e.g., Baluch et al. 2012, Varenhorst et al. 2015a). This mechanism requires a subset of the herbivores population that is virulent, capable of surviving on the resistant genotype of the host plant. By obviating the resistance through a physiological change to the plant, avirulent subpopulations can now survive on the resistant plant. Both mechanisms allow subpopulations that vary by genotype (i.e., virulent and avirulent) to coexist on resistant host plants. These mechanisms have been observed in populations of soybean aphids when colonizing soybean plants that have resistance to soybean aphids (O’Neal et al. 2018, Varenhorst et al. 2015a). Field surveys in North America have observed that soybean aphid biotypes can co-occur in the same fields (Cooper et al. 2015, Alt et al. 2019). Laboratory studies have also shown that virulent and avirulent biotypes can coexist on a shared plant for at least 2–3 generations (Varenhorst et al. 2015a, b, c). However, there is no empirical evidence that soybean aphid biotypes can co-occur on the same plant throughout a growing season.

Exploring the potential for aphid biotypes to coexist on a shared plant is challenging with empirical studies, as several abiotic and biotic factors affect aphid population dynamics. The occurrence and impact of these factors can vary throughout a growing season leading to a local extinction. A thorough empirical study of the season-long potential coexistence of virulent and avirulent aphids would have to address these factors, as well as the many possible situations that could lead to a plant being coinfested with both biotypes. Such a series of experiments would become challenging to conduct. Modeling allows for a rigorous and expansive exploration of biological systems that would otherwise be too expensive or challenging to conduct with empirical-based experiments (Tonnang et al. 2017). Therefore, we elected to modify existing population models developed for aphids on their summer hosts, so that the impact of initial conditions could be explored. Included within this expanded model is resistance to aphids in the host plant, and features that reduce this resistance consistent with feeding facilitation and obviation of aphid resistance. We calculated population growth rates for both virulent and avirulent aphids on susceptible and resistant plants, and use these parameters within this model. The use of this model allowed us to determine what scenarios are likely to result in both biotypes persisting on a shared host plant. The model was used to determine if a ‘within-plant’ refuge is possible, such that avirulent aphids can persist on a resistant plant throughout a growing season. Finally, we discuss how the addition of a third trophic layer (i.e., natural enemies) may affect the outcome of this model and its implications for the implementation of an insect resistance management (IRM) program.

## Material and Methods

### Model Development From a Single Biotype

Kindlemann et al. (2010) are among the first to propose a model describing the population dynamics of aphids using a set of differential equations (Model 1).


$$\begin{array}{l} \frac{dh}{dt}=ax\;;\;h\left(0\right)=0 \end{array}$$

$$\begin{array}{l} \frac{dx}{dt}=\left(r-h\right)x\;\;;\;x\left(0\right)=x_{0} \end{array}$$

Where *h* (*t*) is the cumulative population density of a single aphid biotype at time *t*; *x*(*t*) is the population density at time *t*, *a* is a scalar constant, and *r* is the growth rate of the aphids. The aphid population initially rises due to the linear growth term, but as the cumulative density becomes greater than the growth rate *r*, the population starts to decrease, due to the effects of competition. This results in a hump-shaped population density over time, typical of a boom-bust type scenario (Kot 2001). This is an apt description of aphid dynamics, particularly when exploring soybean aphid dynamics on soybeans during the growing season. The type of population growth described by this model (Kindlemann et al. 2010) has been observed in soybean aphids in North America (e.g., Brosius et al. 2007, Catangui et al. 2009), with colonization in June, then a gradual buildup of population, peaking in August, and declining with aphids dispersing in September to their overwintering host.

Model (1) is quite different from the classical logistic growth model, which predicts growth to a certain carrying capacity. It is an example of a nonautonomous model, wherein the right-hand side of the differential equation depends explicitly on time. The rigorous mathematical analysis of such systems is quite involved, and the methods of classical autonomous systems do not apply (Langa et al. 2002). Hence the rigorous dynamical analysis of model (1) is not found elsewhere. However, it provides a starting point to model more intricate aphid dynamics, particularly when a species presents two or more biotypes.

### Virulent and Avirulent Aphids: Two Biotypes

Subpopulations of a herbivorous species can be organized into biotypes, defined as genotypes capable of surviving and reproducing on host plants containing traits (e.g., antibiosis and or antixenosis) conferring resistance to that herbivore (Downie 2010). Specifically, for the soybean aphid, biotypes are classified based on their ability to colonize soybean varieties expressing *Rag*-genes (*Rag* is derived from the expression, resistance to *Aphis glycines*). For example, soybean aphid biotype 1 is susceptible to all *Rag*-genes, therefore it is called avirulent. Biotype 2 is virulent to *Rag1* (Kim et al. 2008), biotype 3 is virulent to *Rag2* (Hill et al. 2010), and biotype 4 is virulent to both *Rag1* and *Rag2*, capable of surviving on plants with these genes either alone and together (Alt and Ryan-Mahmutagic 2013). These four soybean aphid biotypes have been found throughout the soybean-producing areas of the Midwest United States (Cooper et al. 2015, Alt et al. 2019).

The model proposed by Kindlemann et al. (2010) cannot describe aphid population dynamics on a soybean plant colonized by both virulent and avirulent aphids. This is because that model does not account for competition or cooperation between the two biotypes. First, the virulent and avirulent are in direct competition for space, similar to interspecies competition. The virulent aphids are also in competition for space with other virulent aphids, as avirulent aphids are in competition for space with other avirulent aphids, a case of intraspecies competition. This interaction can produce competition through direct effects. Furthermore, on a resistant plant, both the avirulent and virulent aphids are able to weaken the plant’s defenses via feeding facilitation. However, for the avirulent aphid this only occurs if it arrives in sufficiently large numbers (Varenhost et al. 2015a). Thus, there is a definite resistant level in the plant that is dependent on the initial density of colonizing avirulent aphids. If the avirulent aphids arrive in sufficient numbers above this level, they could colonize a resistant plant. On the other hand, the virulent biotype alters the plant by obviating the resistance (Varenhost et al. 2015a, b), allowing both virulent and avirulent aphids to survive on *Rag1+2* plants. The suppression of the plant’s resistance level by the virulent biotype, eases the colonization process for the avirulent biotype, which is an indirect form of cooperation at play. Thus, the plant’s resistance is a dynamic process, dependent on the presence and densities of these biotypes.

In model (2), our goal is to simulate a system that considers two soybean aphid biotypes (virulent and avirulent) that are attempting to colonize a soybean plant containing aphid resistance in the form of *Rag* genes. The model includes all of the earlier mentioned interactions and considers a dynamic aphid-resistance level in the plant that is affected by the density of the aphids, in the capacities mentioned earlier.

The expanded model (2) is as follows:


$$\begin{array}{l} \frac{dh}{dt}=a(x_{A}+x_{V})\;\;\; \end{array}$$

$$\begin{array}{l} \frac{dx_{A}}{dt}=(r-h)(x_{A}-R) \end{array}$$

$$\begin{array}{l} \frac{dx_{V}}{dt}=\left(r-h\right)x_{V} \end{array}$$

$$\begin{array}{l} \frac{dR}{dt}=-(k_{r}x_{V}+k_{f}x_{V}+k_{f}sgn\left(x_{A}-A\right)x_{A})R \end{array}$$

Here *x*_*A*_(*t*) refers to avirulent aphid population density and *x*_*V*_(*t*) refers to the virulent aphid population density, *h* is the combined cumulative population density of both avirulent and virulent aphids, respectively, at time *t. r* is the maximum potential growth rate of the aphids. *a* is a scaling constant relating aphid cumulative density to its own dynamics. *R* is the dynamic resistance threshold of the plant (Berec 2004). This decreases due to both avirulent and virulent aphid density, that is *x*_*V*_ and *x*_*A*_. It is measured in the same units as aphid density. *k*_*f*_ is the rate of feeding facilitation and *k*_*r*_ is the rate of obviation of resistance (Varenhorst et al. 2015a). Feeding facilitation occurs for avirulent aphids, if their population is above a threshold level *A,* below this threshold, the effect of feeding facilitation by an avirulent population is negligible. Previous studies have demonstrated that obviation of resistance is much more effective in suppressing the resistance than feeding facilitation (Varenhorst et al. 2015a). Therefore, *k*_*r*_ > *k*_*f*_ whenever both the effects take place simultaneously. *Sgn* (*x*_*A*_*−R*) is a Boolean function returning 0 or 1. It returns 1 if the input is strictly positive (i.e., (*x*_*A*_−*R*) > 0) or else it returns 0. This function regulates whether avirulent aphids have enough initial population density to induce the effect of feeding facilitation on the plant.

### Model Parameters

We explored a time series analysis of the soybean aphid population dynamics on a soybean plant containing aphid resistance in the form of *Rag1+2* genes. We used values in the model based on our understanding of the dynamics between the two biotypes. One of the most important parameters of our model is the growth rate of the aphids (*r*). This determines the timing of the boom-bust scenario along with the cumulative population density. The growth rate *r* of the biotypes on resistant (*Rag1+2*) and susceptible plant was estimated using a life table analysis. Treatments consisted of two factors, soybean cultivar (susceptible and *Rag1+2*) and aphid biotypes (avirulent and virulent). Soybean aphids used in this experiment have been kept at Iowa State University, reproducing parthenogenically on soybeans (V3–V7 growth stage). Aphids were kept in separated growth chambers under controlled conditions [25 ± 2°C, 70% RH, and a photoperiod of 16:8 (L:D) h]. The avirulent aphid was reared on a susceptible soybean (LD14-8007), while the virulent was reared on a *Rag1+2* soybean variety (LD14-8001). Soybean seeds were sown in 8-cm-diameter plastic plots using a soil mixture (Sungro Horticulture Products, SS#1-F1P, Agawam, MA). Plants were kept in a greenhouse [25 ± 5°C and a photoperiod of 16:8 (L:D) h], watered three times per week, and fertilized weekly after emergence (Peters Excel Multi-Purpose Fertilizer, 21-5-20 NPK).

Twenty-four hours before the beginning of the experiment, a single mix-aged, apterous adult aphid was transferred onto a soybean leaflet kept in a Petri dish within a growth room [25 ± 2°C, 50% RH, and a photoperiod of 16:8 (L:D) h]. This allowed us to synchronize the age of the aphids (≤24 h old). A total of 40 adult aphids were used for each avirulent and virulent soybean aphid. After 24 h, a single first instar nymph was transferred to the first trifoliate leave of a V2 (Fehr et al. 1971) soybean plant. Plants were then covered with a mesh net to prevent aphids from escaping and moving to another plant. These plants were kept in a growth room [25 ± 2°C, 50% RH, and a photoperiod of 16:8 (L:D) h], and watered three times per week. Each treatment combination consisted of 25 potted plants.

Evaluations were performed daily until the aphid died. Morphological characteristics were used to determine the growth stage (Zhang 1988, Voegtlin et al. 2004) and exuviae were removed once detected. When the aphids became adults, their offspring were counted, and removed daily until the aphid died. All the aphids used in the treatment combination of avirulent aphid and *Rag1+2* variety died within 3 d and were not included in the statistical analysis. Biological and demographic parameters were calculated using the TWOSEX_MSChart (Chi 2020) program following the age-stage, two-sex life table theory (Chi and Liu 1985, Chi 1988). Means and standard error of population parameters were estimated using a bootstrap procedure (Huang et al. 2013) with 100,000 replicates. Differences among treatments were analyzed using a paired bootstrap test at a 5% significant level using the TWOSEX-MSChart program.

## Results

### Parameters Used in the Model

We adjusted the parameters within our model to explore several scenarios in which both virulent and avirulent aphids colonized a single plant. These scenarios are outlined in Table 1. The biological and demographic parameters of avirulent and virulent soybean aphids are presented in Table 2. The treatment consisting of avirulent aphids on a resistant plant was not included in the analysis because all the avirulent aphids died within three days. The intrinsic rate of the increase generated from the life table analysis was used as the growth parameter (*r*) in our model. Life table results showed no significant differences in the intrinsic rate of increase (*r*) between virulent aphids reared on susceptible and resistant soybeans and between avirulent aphids on susceptible soybean and virulent aphids on resistant soybean variety. Because our model does not intend to explore different growth rates for each soybean aphid biotype, the intrinsic rate of increase (*r* = 0.27) used in the model is an average of the three treatments. In our time series analysis, the initial dynamic resistance (*R* (0)) and threshold avirulent population (*A*) are fixed as 30 aphids. The scaling parameter *a* = 0.000005 used in the model comes from Kindlmann et al. 2010. The variables that defined feeding facilitation (*k*_*f*_) and obviation of resistance (*k*_*r*_) were adjusted to explore the impact of each dynamics on the occurrence of the two biotypes as per Table 1. The *k*_*f*_ is taken as 0.001 and *k*_*r*_ as 0.01 whenever not taken as 0 to study the dynamics of feeding facilitation and obviation of resistance as per Table 1.

**Table 1.** Model configuration and its relationship to the results

Feeding facilitation and obviation of resistance	k_f_	k_r_	Notes on the biology and ecology
No feeding facilitation or obviation of resistance	0	0	This represents a plant whose resistance is static, regardless of the plants age or herbivore genotype or density.
Only feeding facilitation	+	0	This occurs when a plant whose resistance is dynamic is feed upon by an avirulent biotype.
Only obviation of resistance	0	+	This scenario is not explored as the absence of feeding facilitation (i.e., *k*_*f*_ = 0) occurs for only a brief period when the plant is infested with a low density of virulent aphids. As that population grows, feeding facilitation will occur.
Both feeding facilitation and obviation of resistance	+	+	This situation occurs when the plant is infested with virulent alone or with both virulent and avirulent biotypes.

**Table 2.** Life table analysis of avirulent and virulent soybean aphids on different plant genotypes

Biological parameter	Avirulent aphid on susceptible soybean	Virulent aphid on susceptible soybean	Virulent aphid on resistant soybean
N1 (days)	2.42 ± 0.12a	1.79 ± 0.08b	2.04 ± 0.11b
N2 (days)	1.75 ± 0.12a	1.54 ± 0.10a	1.54 ± 0.13a
N3 (days)	1.5 ± 0.12a	1.43 ± 0.10a	1.29 ± 0.09a
N4 (days)	1.57 ± 0.10a	1.41 ± 0.10a	1.52 ± 0.10a
APOP (days)	0.33 ± 0.10a	0.19 ± 0.10a	0.34 ± 0.09a
TPOP (days)	7.57 ± 0.1a	6.38 ± 0.14b	6.78 ± 0.17b
Oviposition period (days)	10.90 ± 1.14a	11.33 ± 1.07a	10.34 ± 1.19a
Adult longevity (days)	12.35 ± 1.21a	13.59 ± 1.32a	12.61 ± 1.31a
Fecundity (no. nymphs/female)	26.17 ± 1.35a	30.05 ± 3.62a	27.09 ± 3.66a
Demographic parameter			
Net reproductive rate (Ro)	24.08 ± 3.74a	26.44 ± 3.72a	24.92 ± 3.66a
Finite rate of increase (λ, d^−1^)	1.28 ± 0.01b	1.33 ± 0.01a	1.31 ± 0.01ab
Intrinsic rate of increase (r, d^−1^)	0.25 ± 0.01b	0.29 ± 0.13a	0.27 ± 0.01ab
Mean generation time (T, days)	12.74 ± 0.21a	11.22 ± 0.25b	11.82 ± 0.21b
GRR	39.65 ± 4.30a	40.09 ± 2.87a	39.26 ± 3.40a

Different letters within the same row indicate significant differences (*P* < 0.05) among treatments. APOP, adult pre-oviposition period; TPOP, total pre-oviposition period; GRR, gross reproductive rate.

### Feeding Facilitation

**Fig. 1.**
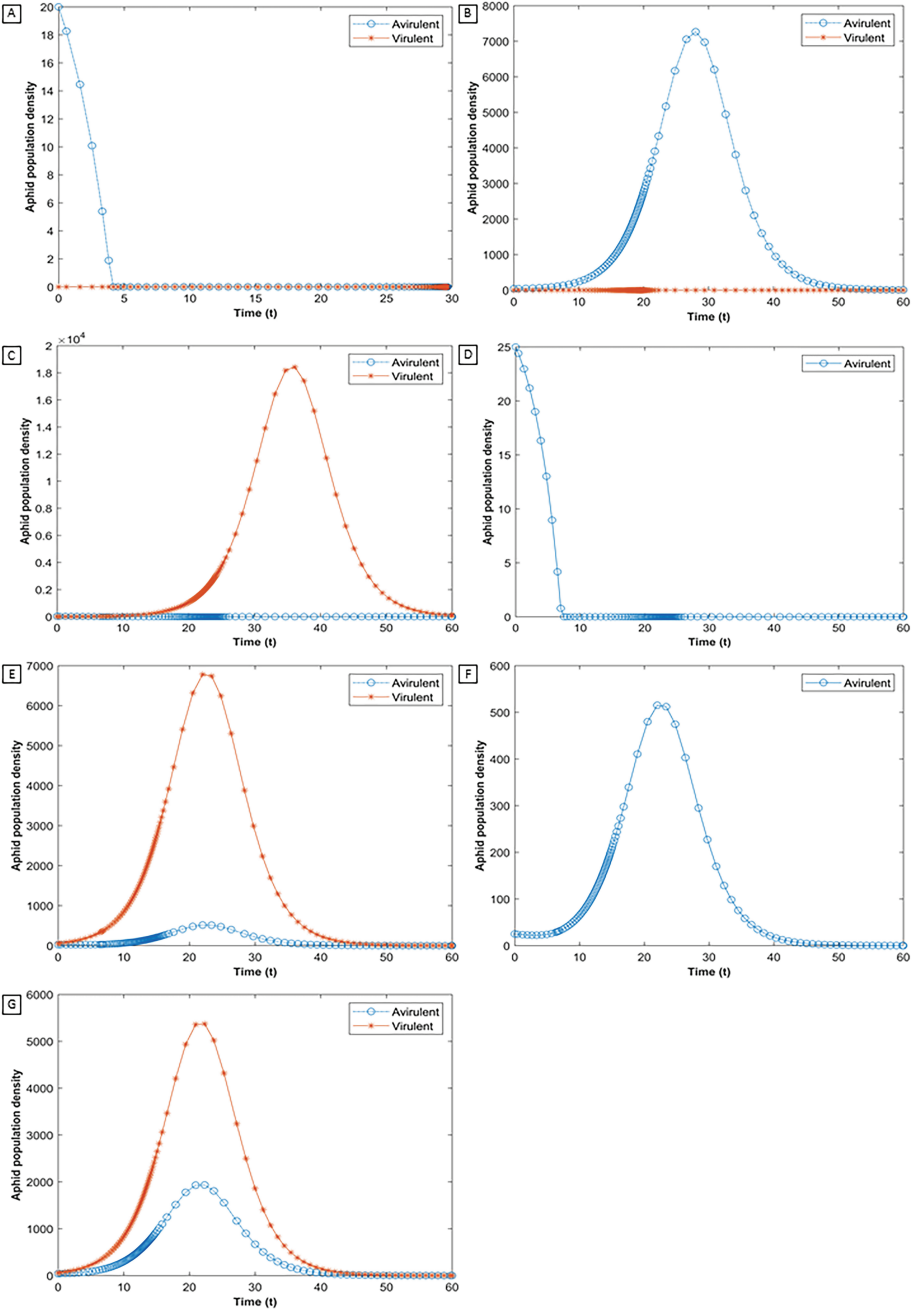
The impact of varying the initial population of the avirulent aphid (*x*_*A*_), the virulent aphid (*x*_*V*_) and the level of aphid resistance in the host plant (*R*) are explored in (A–E). The initial populations of both biotypes are (A) *x*_*A*_(0) = 20 and *x*_*V*_ (0) = 0; (B) *x*_*A*_(0) = 40 and *x*_*V*_ (0) = 0; (C) and (D) *x*_*A*_(0) =25 and *x*_*V*_ (0) = 5; (E) and (F) *x*_*A*_(0) = 25 and *x*_*V*_ (0) = 60; (G) *x*_*A*_(0) = 40 and *x*_*V*_ (0) = 60. Results reported in (A and C–D) demonstrate that the model accounts for the impact of resistance on an avirulent population, (B and E–G) demonstrates the capacity for avirulent aphids to overcome this resistance. In (E–G), the virulent aphids reach a higher peak population than the avirulent aphids, with an increase in their initial population. In (C and D) avirulent aphid goes to extinction while virulent aphid reaches a high peak.

While studying feeding facilitation within the model on an aphid-resistant plant, we removed the effect of obviation of resistance due to virulent aphids by setting *k*_*r*_ = 0. The model shows that when virulent aphids are absent and the initial avirulent population is below the resistance threshold, the avirulent aphid goes to extinction as feeding facilitation does not take place due to a low initial population (Fig. 1A). However, when the population of avirulent aphid is above the resistance threshold, colonization takes place even in the absence of the virulent aphid (Fig. 1B). When the initial population of virulent aphids is very small and the population of avirulent aphids is below the resistance threshold, the effect of feeding facilitation is insufficient to allow for the coexistence of both biotypes (Fig. 1C and D). Even in the absence of resistance obviation, if the initial population of virulent aphids is increased, the model accounts for this increase in the overall population of aphids on the plant by increasing the strength of feeding facilitation. This further sustains avirulent aphids on the plant throughout the season even when the initial avirulent aphid is below the resistance threshold (Fig. 1E and G).

A significant increase in the peak population of avirulent aphid is observed with an increase in their initial population. This relationship is apparent when comparing Fig. 1E and G, as an increase in the initial population of the avirulent aphid results in a higher maximum population. The model fixes the value of the host plant as a resource for aphids. Thus, an increase in the avirulent population results in a decrease in the maximum population attained by virulent aphids in the season.

### Obviation of Resistance

**Fig. 2.**
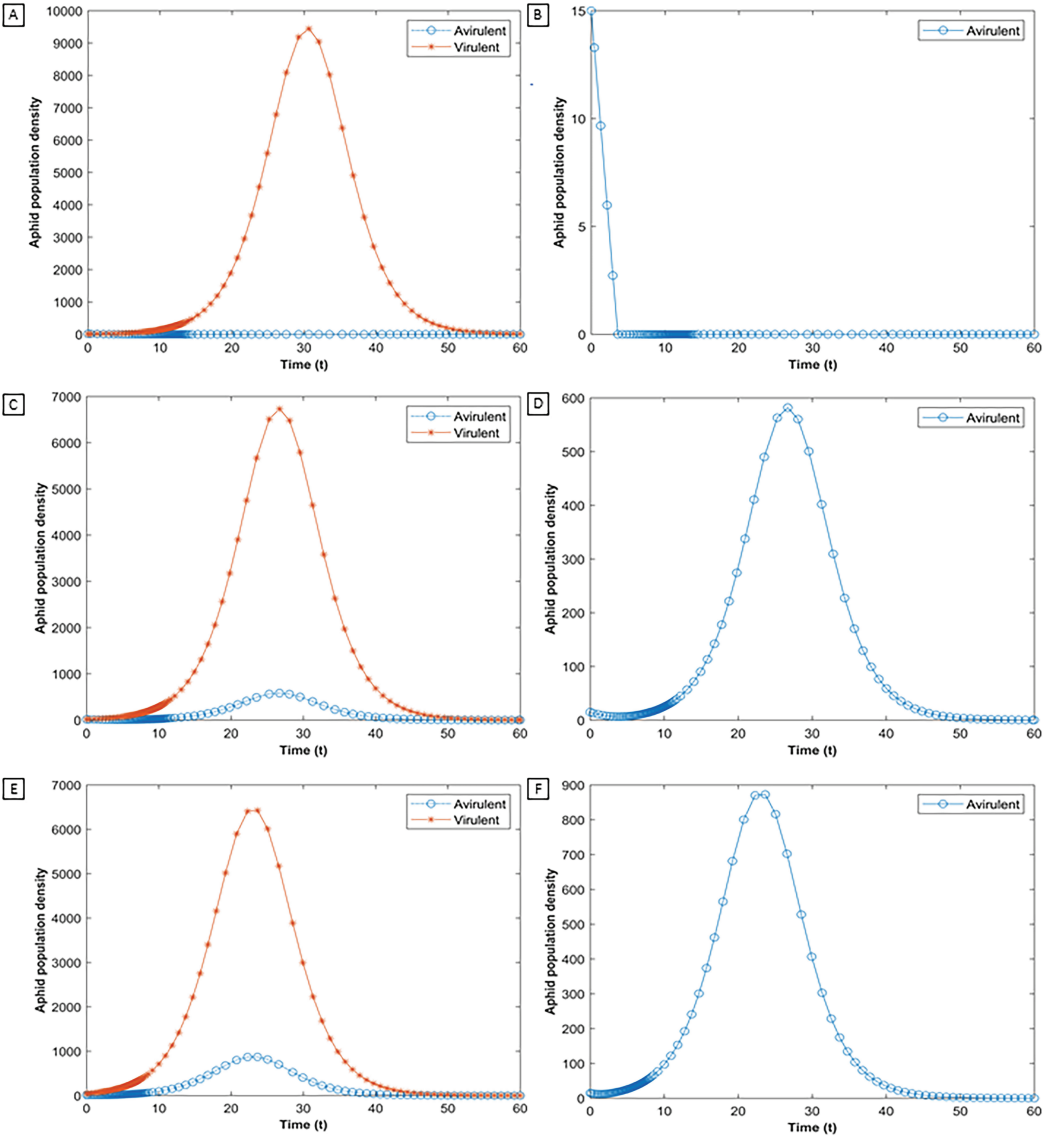
The impact of increasing the initial population of the virulent aphid (solid line with asterisk marker, *x*_*V*_) on the co-existence of virulent and avirulent aphids (solid line with circular marker, *x*_*A*_) on a single, aphid-resistant soybean plant (*R*(0) set to 30) are explored in (A, C, and E). Figures (B, D, and F) are the same figures as (A, C, and E) respectively showing the zoomed in view of only the avirulent aphids. The initial populations of both biotypes are (A and B) *x*_*A*_(0) = 15 and *x*_*V*_ (0) = 10; (C and D) *x*_*A*_(0) = 15 and *x*_*V*_ (0) = 20; (E and F) *x*_*A*_(0) = 15 and *x*_*V*_ (0) = 50. In all the scenarios modeled, the virulent aphid outcompetes the avirulent aphid. However, as the initial population of virulent aphids increases, so too does the peak population of avirulent aphids. In figures (B, D, and F), the population of avirulent aphids is reported alone for each of the three scenarios, revealing their phenology across the modeled growing season. Note that the avirulent population went extinct early in the season when the initial population of virulent aphids was at its lowest (B).

Obviation of resistance is a phenomenon by which the virulent aphids suppress the resistance of the resistant plant, allowing both the virulent and avirulent aphids to colonize and grow on a shared plant. In Fig. 2, we explored if our model could produce results consistent with this phenomenon. We set the initial population of the avirulent aphid lower than the resistance threshold in all the cases, while holding the initial avirulent population and the resistance threshold constant in all cases. This prevents feeding facilitation, as noted in the previous section.

As shown in Fig. 2A and B, when the virulent aphid population is very low (i.e., below *A*), the resistance is not yet suppressed and the avirulent aphid goes extinct. By increasing the initial virulent aphid population from 20 to 50, the avirulent aphid persists on the plant (Fig. 2C and D). There was a sufficient density of virulent aphids in these scenarios to suppress the resistance in the plant, allowing the avirulent aphid to survive on the resistant plant. As we increase the initial population of virulent aphid (Fig. 2E and F), the resistance declines much faster allowing the avirulent aphid to reach higher densities.

Obviation of resistance as described in this model allows for the persistence of the avirulent aphid at population levels lower than what the resistance threshold would allow. With an increase in the initial virulent aphid, obviation of resistance begins sooner, resulting in higher populations of avirulent aphid across the modeled season. We also see that the closer the initial virulent aphid population is to the resistance threshold, the fewer initial virulent aphids are required to obviate resistance such that the avirulent aphid population is sustained.

### Coexistence of Two Biotypes

**Fig. 3.**
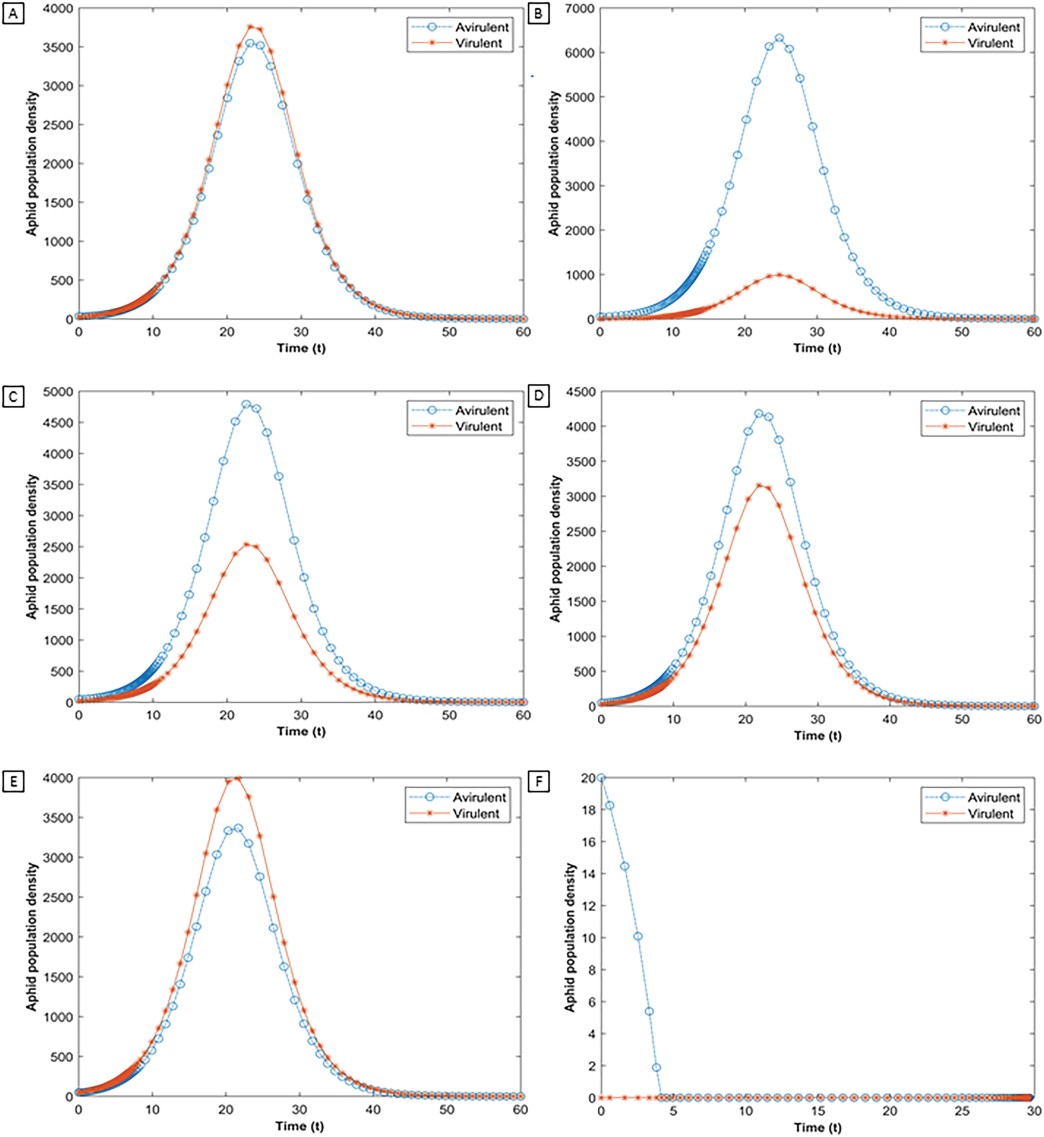
The impact of both aphid biotype population over time with varying initial population on an aphid-resistant plant. Soybean aphid dynamics when the initial populations of avirulent virulent aphid are greater than the resistance level. The initial populations of both biotypes are: (A) *x*_*A*_(0) = 35 and *x*_*V*_ (0) = 25; (B) *x*_*A*_(0) = 50 and *x*_*V*_ (0) = 5; (C) *x*_*A*_(0) = 50 and *x*_*V*_ (0) = 20; (D) *x*_*A*_(0) = 50 and *x*_*V*_ (0) = 30; (E) *x*_*A*_(0) = 50 and *x*_*V*_ (0) = 50; (F) *x*_*A*_(0) = 15 and *x*_*V*_ (0) = 0. The initial resistance of the plant is same in all figures (R (0) = 30). In the figures from (A–E) both biotypes persist together throughout the season. In figure (F) the avirulent aphid is unable to persist on the plant due to lack of virulent aphid population.

The coexistence of both biotypes can be seen on a resistant soybean plant. Varying the initial population of the biotypes enables sustenance of the avirulent aphid on the plant, due to the reduction of resistance by the virulent aphid resulting from obviation of resistance and feeding facilitation. If the initial avirulent population is higher or equal to the resistance threshold then the two aphid biotypes coexist for the whole season (Fig. 3A–E). When initial population of avirulent aphid is set higher than the resistance threshold (Fig. 3A–E) both biotypes can persist on the plant regardless of initial virulent aphid density. We also observe that if initial avirulent population is low but close to the resistance threshold then both populations co-exist (Fig. 2C–F). Which population will dominate is dependent on the initial density of each biotype. For example, an increase of the virulent aphid’s initial population results in a larger population density compared with the avirulent aphid population. Nonetheless, the peak for the population of both biotypes occurs at the same time. In summary, a soybean plant that is coinfested with virulent and avirulent aphids at the same time can lead to both biotypes being present on the plant at the end of the growing season as seen, before the overwintering process begins – thus there is the possibility of a ‘within plant refuge’. The simulation results are dependent however on the initial populations of the aphids and whether they are significantly above or below the resistance threshold of the plant.

## Discussion

The model accounts for the dynamics of feeding facilitation with both avirulent and virulent soybean aphids. If the plant is resistant (i.e., it contains *Rag*-genes), then virulent aphids survive and feeding facilitation takes place at any population level. If this resistant plant is colonized by avirulent aphids only, survival is not guaranteed. However, if population of avirulent aphids is higher than the resistance threshold (*R*), feeding facilitation (Varenhorst et al. 2015a) allows this biotype to survive on a resistant plant.

This model is studying antibiosis, a specific mechanism of plant resistance to an insect herbivore. This is only one of three mechanisms that have been exploited by plant breeders to develop commercial cultivars by which plants avoid the impact of herbivory on their fitness or fecundity (Painter 1951). Both antixenosis and antibiosis are conferred by *Rag*-genes, with the specifics of these mechanisms yet to be fully described. To date, antibiosis is considered the primary mechanism by with soybean plants are protected from soybean aphids when both *Rag1* and *Rag2* are present (Natukunda and MacIntosh 2020). There is evidence that isoflavones can have a deterrent effect (i.e., antixenosis) on soybean aphids (Hohenstein et al. 2019), and may contribute to antibiosis as well (Meng et al. 2011, Joshi et al. 2021), but this has not been confirmed for *Rag1+2* containing soybeans. Regardless of the specific mechanism, empirical evidence across multiple, repeated studies has revealed that virulent aphids systemically shut down this resistance such that avirulent aphids can survive (Varenhorst et al. 2015a, b, c). There is clearly a substantial amount left unknown about the suborganismal aspects of how soybean aphids obviate *Rag*-based resistance. Regardless of how soybean aphids obviate this resistance, the impact of a virulent aphid colonizing a resistant plant persists for at least 14 d of a coinfestation by an avirulent population (Varenhorst et al. 2015a). By modifying a relatively simple model for describing aphid populations, we demonstrated that this coinfestation could persist throughout a growing season. We note that this may not be true for soybean plants for whom the resistance is more than just antibiosis.

Currently, the frequency of virulent biotypes within North America is lower than that of avirulent biotypes (Cooper et al. 2015, Alt et al. 2019). This scenario is necessary for *Rag*-based resistance to remain useful for the management of this pest. With the increased use of a *Rag*-resistance, the frequency of virulent biotypes is expected to increase. Efforts to prevent virulent biotypes from increasing with increasing use of aphid resistance are the goal of an IRM program. A strategy for the sustainable use of a resistant variety is the creation of a ‘refuge’ of susceptible plants that maintain a sufficient population of avirulent biotypes to reduce the frequency virulent biotypes in subsequent generations (Crowder and Carriere 2009). By coupling a refuge with resistant plants that are so toxic that the virulence becomes functionally recessive, this strategy has preserved the value of insect-resistant plants (Tabashnik et al. 2013). A challenge to this strategy is farmer acceptance of a practice that requires the cultivation of plants susceptible to the target pest. Refuges have been incorporated into the units of seed sold to farmers so that this strategy is practiced with limited input from the farmer. This practice has been referred to as a ‘refuge-in-a-bag’. The soybean aphid/*Rag*-resistance system suggests that a refuge could occur within a plant. For this refuge to contribute to the management of virulent populations in an IRM program, the avirulent populations must persist throughout the growing season and contribute to the population that returns to the overwintering host.

This model suggests that there is the potential to contribute to such a refuge from the limited empirical evidence of feeding facilitation and the obviation or resistance. The full potential of a ‘refuge-in-a-plant’ requires more empirical evidence that the avirulent populations generated from a within plant refuge contribute to the overwintering population. The evidence that virulent and avirulent aphids can coexist on resistant plants has been observed within laboratory microcosms and caged plants within a field setting (Varenhorst et al. 2015a, b, c). There is an absence of evidence to support the result from the simulations which show cooccurrence for a period of 60 d, which is a period of time that includes the arrival and colonization through the senescing stage of the soybean plant to the beginning of the overwintering process. These simulations give us explicit information about the initial populations of the virulent and avirulent aphids which can be further explored in future lab and field experiments to confirm that biotypes can coexist on soybeans. The results from the previously published empirical studies and this model are consistent with a more general model that predicts the success of a weaker competitor utilizing a shared resource (Parshad et al. 2021). Combined, these models suggest that invasive insect herbivores can be managed by manipulating the conditions by which they compete on a shared host plant (Parshad et al. 2021).

An additional detail that would improve our modeling efforts is the role of natural enemies in affecting the frequency of virulence. Natural enemies can affect the frequency of biotypes that are virulent to resistant host plants (Gould et al. 1991). A community of aphidophagous predators can be found in soybean fields that feed on soybean aphids during the summer. Studies manipulating aphid exposure to natural enemies using cages demonstrated that predators play a role in suppressing the growth of aphid populations in North America (Costamagna and Landis 2006, Costamagna et al. 2007, Bannerman et al. 2018). Parasitoids are an additional source of soybean aphid mortality that can significantly impact population growth especially in their native range (Liu et al. 2004). Recent efforts in North America to release parasitoids from Asia have shown initial signs of success (Frewin et al. 2010, Kaser and Heimpel 2018). Going forward, it is unclear what impact the combination of predators and parasitoids may have on soybean aphid populations within North America. Modeling by Chang and Kareiva (1999) suggests that both types of natural enemies can significantly limit the population growth of an herbivore, like an aphid. However, the impact of either depends upon the initial population of the given natural enemy and its efficiency in killing the aphid prey. For example, a relatively large population of less efficient predators can be as effective as a lower population of more efficient parasitoids. We note that the model developed by Chang and Karieva (1999) does not address the combined impact of predators and parasitoids. Such a modeling effort may appear simply additive but empirical data is needed to determine if intra-guild predation between the two natural enemies limits their combined impact. Furthermore, it is not known if the impact of either predators or parasitoids varies significantly by aphid biotype. For example, does a given natural enemy have a preference for a virulent or avirulent aphid? Such data is required to more accurately model the soybean aphid-soybean system. Revealing the impact of natural enemies on the dynamic relationship between biotypes on aphid-resistant plants may be critical for developing an IRM strategy for soybean aphids.
